# Getting to the genetic and environmental roots of educational inequality

**DOI:** 10.1038/s41539-018-0021-1

**Published:** 2018-03-23

**Authors:** Nicholas Martin

**Affiliations:** QIMR Berghofer Medical Research Institute, Brisbane, QLD Australia

Few topics excite more comment and controversy than the causes of inequality in educational outcomes that are manifest all about us; this often spills over into polarised political positions and sometimes bitter debate with catch-phrases of 'class privilege' and 'social deprivation' being thrown from one side to the other, unhampered by much in the way of critical evidence. A circuit breaker to this stale, repetitive argument comes from an entirely unexpected quarter—molecular biology coupled with some clever statistical genetics.

The sequencing of the first human genomes around the turn of the millennium cost millions of dollars each and there was despair that this technology could never be extended to large samples. However, it turns out that the genome is organised into haplotype blocks within which there is a very high degree of correlation (linkage disequilibrium—LD, hence LD blocks) between adjacent gene variants (single nucleotide polymorphisms, or SNPs).^1,2^ In layman’s terms, this means that genes that lie close to each other on a chromosome tend to be inherited together.

You can get most of the information about a person’s genome (up to 95%) by genotyping for few SNPs that are associated with specific regions of the genome called LD blocks. SNPs are the most common type of genetic variation among people, and genotyping just a few SNPs per LD block, perhaps 700,000 in all, on a commercial 'SNP chip' is relatively inexpensive (less than $50 per genome). Whereas, sequencing the whole 3.3 billion bases in the genome would cost (factoring in essential bioinformatics post-processing fees) around $5000—that is, you can SNP chip about 100 people for the same cost as full sequencing of a single individual.

Genomic sequencing is the ideal tool for rare (Mendelian) genetic diseases determined by a single gene of very large effect (e.g., cystic fibrosis or Tay-Sachs disease), where only a few individuals need to be sequenced to detect the causative mutation. But for complex diseases and traits (e.g., asthma, schizophrenia, height, cognition) it turns out that hundreds or thousands of genes are involved, each of individually small effect size, but collectively accounting for 20–80% of variance of most complex traits. This means that very large samples (tens, hundreds of thousands) of genotyped individuals are needed to detect the causal variants. However, even if SNPs do not reach the very stringent formal level for individual significance (*p* = 0.05/1,000,000 = 5e-8), their effects can be combined in a simple linear regression equation to create a polygenic risk score (PRS), the sum of all SNP effect sizes weighted by their frequencies. Basically, if a specific set of SNPs is found to be associated with a particular trait more frequently than would be expected by random chance, it is more likely that those SNPs influence that trait. Thus PRS, derived from a large discovery sample, has proved remarkably successful at predicting variance in the same trait (and sometimes related traits) in entirely independent smaller (target) samples.

Watching the increasingly spectacular success of medical scientists in the genome wide association studies (GWAS) revolution and hundreds of SNPs being discovered significantly associated with traits as diverse as lipid levels and age at menarche, social scientists began to wonder whether their favourite variables might also yield to this breakthrough technology. Educational attainment (EA) was the first candidate, since it is collected as a standard sociodemographic covariate in most studies, and most importantly in the disease GWAS studies where subjects had already been genotyped; so to contribute EA GWAS data to an international meta-analysis consortium was simple.

The first EA GWAS consortium paper (EA1) was published in *Science* in 2013 with 126,559 subjects.^3^ Two metrics were used—years of education on a 4-point scale (carefully calibrated to take account of different educational systems in different countries), and the second much simpler, a binary variable 'Been to college (in the American sense) yes/no'. They found 3 significant SNPs and accounted for ~2% of variance in independent samples. The second paper (EA2) was published in *Science* in 2016, now with 293,723 subjects, 74 significant SNPs, and accounting for ~4% of variance in independent samples.^4^ Most interestingly, the authors reported high genetic correlations between SNPs that influence EA and those that influence brain volume, IQ, and several other obvious correlates. A third study, EA3, was presented at a conference in June 2017 with *n* > 700,000, ~550 hits, and 10% variance accounted for. Publication of this is being held until the new UK Biobank tranche of 460,000 subjects has been added, which will make this the largest GWAS sample ever assembled of over 1 million subjects, which should provide a really powerful predictive instrument.

Smith–Woolley and colleagues have made use of the published EA2 results to examine the vexed question of large mean differences in exam performance between the three largest groupings of UK secondary schools—state schools that are selective in their entrance requirements (grammar schools), state schools that are not selective, and private schools that variably exercise some degree of selection, either directly or indirectly.^5^ Using their large longitudinal twin sample (TEDS) and analysing their results for the GCSE (General Certificate of Education) at age 16, the authors find the usual large mean differences between school types, that is, non-selective schools doing worst, grammar schools doing best, and with private schools a little below grammar schools.

Because the TEDS sample has been genotyped, it is possible to calculate PRS for them all, using the weights from EA2 (to which TEDS did not contribute). Mean EA2 PRS between the school types follows the same pattern as the GCSE scores, the grammar schools’ mean, being 0.44 standard deviations above that of unselected schools, and for private schools, 0.37sd above the unselected mean (the difference between these latter two not significant). When the GCSE grades are corrected for the EA2 PRS, the gaps are lessened but not removed, as they are also by correction for socioeconomic status (SES) and, most notably, prior achievement (which is hardly surprising). When all selective criteria used to determine who went to which school type were jointly taken into account the mean differences between schools largely disappeared.

The key conclusion is that mean differences between GCSE performance between school types are largely due to selective factors as to who gets in, or not, and further, that these are to a considerable extent determined by genetic factors partly measurable by EA PRS. Once these factors are taken into account there is little residual variance that might be attributed. Complicating this analysis are the strong genetic correlations between outcome and predictor variables—EA, SES, prior ability, and prior achievement, and disentangling cause and effect between these will be a formidable task. But the authors have made a bold and useful start, and one that will only get more informative as the EA PRS instrument gets stronger with EA3/4 to be published next year.

One can only hope that the lessons of this paper are noticed by our politicians. I refer particularly to a recent Australian Prime Minister who took the fact of the low rate of university entrance in her working class electorate compared with that in the middle class electorate next door as prima facie evidence of social discrimination, and many other politicians have committed similar errors of inference. It would be interesting to estimate the mean EA PRS in the two electorates.

Molecular biology can also cast light on the nature of environmental variance, as manifested in patterns of DNA methylation, one of the primary epigenetic mechanisms for regulating gene expression, which can arise from both endogenous (genetic or stochastic) and exogenous (external environmental) sources. To analyse this, van Dongen and colleagues compared the degree of DNA methylation in peripheral blood at over 400,000 sites across the genome in over 4000 Dutch subjects to their EA, and found significant associations at 58 sites.^6^ The van Dongen study shows that disentangling the biology behind the epigenetic EA candidate loci requires detailed studies of environmental factors influencing the DNA methylome and that these types of studies are more difficult to interpret than GWAS. As the authors stress, EA-associated variation at specific DNA methylation sites may point to three ongoing processes. Firstly, variation may indicate epigenetic consequences of differential life conditions that correlate with educational attainment (i.e., epigenetic biomarkers of exposure). Secondly, variation may represent peripheral correlates of the epigenetic mechanisms that contribute to individual differences in educational attainment, for instance by regulating gene expression in neurons. Thirdly, variation may mark peripheral epigenetic correlates of education-related health differences (which may be biomarkers or may be part of the causal mechanisms that contribute to disease, respectively).

The paper clearly shows that methylation sites associated with educational attainment in blood reveal epigenetic consequences of differential exposures that correlate with educational attainment, including cigarette smoke, air pollution and maternal folate levels. This finding highlights the value of DNA methylation patterns in circulating cells to get an objective measure of differential exposures connected to educational attainment or other characteristics in human populations, and warrants further investigation into the health consequences of EA associated DNA methylation signatures.

The extensive follow-up analyses of this paper will put the findings in a broader perspective, and includes integration with RNA-sequence data, the use of twin data to shed light on the role of genetics and environment in contributing to variation in DNA methylation between people at these loci, and the use of public data on DNA methylation in blood and brain samples. The inclusion of RNA sequencing will help shed light on what effect the DNA methylation levels actually have on gene expression, since DNA methylation can positively or negatively affect gene expression and can influence isoform specific gene expression.

It is noteworthy that this study identified more EA-associated DNA methylation sites in a sample size of 4152, compared to a recently published larger international meta-analysis of educational attainment and whole blood DNA methylation that included 10,767 participants from 27 cohorts from different countries (top findings from the two studies largely overlapped).^7^ This suggests that the homogeneous study population of the van Dongen et al. study (same educational system and social conditions) may have been an advantage.

The overlap between methylation signatures associated with EA and smoking, the fact that some of this signal remains associated with educational attainment after adjusting for smoking, and the observation that DNA methylation level in blood correlates with DNA methylation level in brain tissue at 17% of the 58 sites, generates interesting questions for future studies: which smoking-associated DNA methylation signals are not merely a reflection of smoking exposure (or other pollutants) but are also a driver of smoking behaviour? Does the methylation level of these loci directly influence EA via altered gene expression? The authors suggest Mendelian randomisation analyses using SNPs known to alter methylation at the sites associated with EA, and we look forward to the time when there is sufficient power to perform such analyses.

Meanwhile, there is no doubt that the work presented in both these outstanding papers has taken the discussion of environmental effects on educational attainment to a higher level.
